# Supplementary material for: Comparative genomics provides new insights into the diversity, physiology, and sexuality of the only industrially exploited tremellomycete: Phaffia rhodozyma
Source: BMC Genomics. 2016 Nov 9;17:901. doi: 10.1186/s12864-016-3244-7 (PMC5103461; doi:10.1186/s12864-016-3244-7)
Supplement: Additional file 6: — List of orphan genes with links to PFAM (related to Additional file 1: Table S1). (ZIP 1428 kb) [file 12864_2016_3244_MOESM6_ESM.zip › PFAM_UNIQUE/G01242_P.html]

#                                                                            --- full sequence --- -------------- this domain -------------   hmm coord   ali coord   env coord
  
# target name        accession   tlen query name           accession   qlen   E-value  score  bias   #  of  c-Evalue  i-Evalue  score  bias  from    to  from    to  from    to  acc description of target
  
#------------------- ---------- ----- -------------------- ---------- ----- --------- ------ ----- --- --- --------- --------- ------ ----- ----- ----- ----- ----- ----- ----- ---- ---------------------
  
G01242\_P             -            685 Caldesmon            PF02029.10   492       4.7    4.2   9.3   1   1     0.078       8.3    3.3   9.3   101   195    48   128    18   181 0.42 - Caldesmon  
G01242\_P             -            685 CytadhesinP1         PF12378.3     72      0.13   10.9   3.3   1   2    0.0068        20    3.9   0.3    10    46    39    87    32    90 0.75 - Trypsin-sensitive surface-exposed protein  
G01242\_P             -            685 CytadhesinP1         PF12378.3     72      0.13   10.9   3.3   2   2   0.00099         3    6.6   0.2    42    66   479   503   469   505 0.82 - Trypsin-sensitive surface-exposed protein  
G01242\_P             -            685 MIP-T3               PF10243.4    539      0.95    6.5  21.4   1   1      0.01       1.3    6.1  21.4    99   197    52   149     2   354 0.53 - Microtubule-binding protein MIP-T3  
G01242\_P             -            685 PRP21\_like\_P         PF12230.3    229       6.5    4.8  12.2   1   1     0.019        16    3.5  12.2    93   163    45   113    29   151 0.74 - Pre-mRNA splicing factor PRP21 like protein  
G01242\_P             -            685 Utp14                PF04615.8    735       4.7    3.9  18.1   1   1     0.089       8.3    3.1  18.1   417   533    31   143    13   183 0.48 - Utp14 protein
